# A multicenter, randomized, open-label pilot trial assessing the efficacy and safety of etanercept 50 mg twice weekly followed by etanercept 25 mg twice weekly, the combination of etanercept 25 mg twice weekly and acitretin, and acitretin alone in patients with moderate to severe psoriasis

**DOI:** 10.1186/s12895-016-0048-z

**Published:** 2016-07-25

**Authors:** Joo-Heung Lee, Jai-Il Youn, Tae-Yoon Kim, Jee-Ho Choi, Chul-Jong Park, Yong-Beom Choe, Hae-Jun Song, Nack-In Kim, Kwang-Joong Kim, Jeung-Hoon Lee, Hyun-Jeong Yoo

**Affiliations:** 1 Department of Dermatology, Samsung Medical Center, Sungkyunkwan University School of Medicine, 50 Irwon-dong, Gangnam-gu, Seoul, Korea; 2 Department of Dermatology, National Medical Center, Seoul, Korea; 3 Department of Dermatology, College of Medicine, The Catholic University of Korea, Seoul, Korea; 4 Department of Dermatology, Asian Medical Center, University of Ulsan College of Medicine, Seoul, Korea; 5 Department of Dermatology, Konkuk University School of Medicine, Seoul, Korea; 6 Department of Dermatology, College of Medicine, Korea University, Seoul, Korea; 7 Department of Dermatology, College of Medicine, Kyung Hee University, Seoul, Korea; 8 Department of Dermatology, Hallym University Sacred Heart Hospital, Seoul, Korea; 9 Department of Dermatology, School of Medicine, Chungnam National University, Daejeon, Korea; 10 Pfizer Pharmaceuticals Korea Limited, Seoul, Korea

**Keywords:** Psoriasis, Etanercept, Acitretin, Combination therapy, Efficacy, Safety, Korean patients

## Abstract

**Background:**

Etanercept, a soluble tumor necrosis factor receptor, and acitretin have been shown to be effective in treating psoriasis. Acitretin is widely used in Korea. However, the combination of etanercept plus acitretin has not been evaluated among Korean patients with psoriasis. The objective of this study was to investigate the efficacy and safety of combination therapy with etanercept and acitretin in patients with moderate to severe plaque psoriasis.

**Methods:**

Sixty patients with psoriasis were randomized to receive etanercept 50 mg twice weekly (BIW) for 12 weeks followed by etanercept 25 mg BIW for 12 weeks (ETN-ETN); etanercept 25 mg BIW plus acitretin 10 mg twice daily (BID) for 24 weeks (ETN-ACT); or acitretin 10 mg BID for 24 weeks (ACT). The primary efficacy measurement was the proportion of patients achieving 75 % improvement in Psoriasis Area and Severity Index (PASI 75) at week 24. Secondary end points included 50 % improvement in PASI (PASI 50) at week 24 and clear/almost-clear by Physician Global Assessment (PGA) at each visit through week 24.

**Results:**

The proportions of patients achieving PASI 75, PASI 50, and PGA clear/almost-clear at week 24 in the ETN-ETN (52.4, 71.4, and 52.4 %, respectively) and ETN-ACT groups (57.9, 84.2, and 52.6 %, respectively) were higher than in the ACT group (22.2, 44.4, and 16.7 %, respectively). The incidence of adverse events was similar across all arms. This was an open-label study with a small number of patients.

**Conclusion:**

In Korean patients with moderate to severe plaque psoriasis, etanercept alone or in combination with acitretin was more effective than acitretin. All treatments were well tolerated throughout the study.

**Trial registration:**

This study was registered on July 7, 2009 at ClinicalTrials.gov, NCT00936065.

## Background

Psoriasis is a chronic autoimmune condition that affects 1 to 3 % of the general population worldwide [1–3]. Psoriasis has been associated with an increased risk for arthritis [4, 5], diabetes [4, 6, 7], cardiovascular disease [4, 8], depression [4, 9], and poor quality of life (QoL) [4, 10]. Although there is as yet no cure [1–3], there are several effective treatments available to manage the disease [11–16].

In clinical trials, etanercept, a tumor necrosis factor-alpha (TNFα) inhibitor, has been shown to be effective in managing psoriasis including improvements in Psoriasis Area Severity Index (PASI) scores [17–19], Physician’s Global Assessment (PGA) [18, 19], and QoL [19, 20]. Acitretin, a systemic retinoid, is also effective for the management of psoriasis [21]. Acitretin is frequently used in combination with other agents (e.g., phototherapy and vitamin D), since acitretin monotherapy is often only moderately effective [21–24]. Since acitretin is not an immunosuppressive agent, combination treatment with etanercept may have a synergistic effect with a low risk of toxicity [21, 24]. This has been demonstrated in a pilot study in which the combination of acitretin and low-dose etanercept was as effective as high-dose etanercept, and both were significantly more effective than acitretin alone [25]. The purpose of the current study was to evaluate combination therapy of etanercept plus acitretin among Korean patients with psoriasis.

## Methods

### Patients

This study was reviewed and approved by local Institutional Review Boards (Appendix) and was conducted in compliance with the ethical principles originating in or derived from the Declaration of Helsinki and in compliance with Good Clinical Practice Guidelines. All patients provided signed informed consent.

Patients were eligible for study enrolment if they were at least 18 years of age and had active, clinically stable moderate to severe plaque psoriasis involving ≥10 % body surface area (BSA) or PASI ≥10. Exclusion criteria included evidence of skin conditions (e.g., eczema) other than psoriasis that would interfere with evaluations of the effect of study medication on psoriasis; any rheumatologic disease; prior exposure to biologic therapies, including etanercept, within 24 weeks of baseline visit; previous history of phototherapy or any systemic or topical therapy, including acitretin, for psoriasis within the previous 28 days before baseline visit; uncontrolled hypertension or diabetes mellitus; any severe hematologic, cardiovascular, renal, hepatic, or pulmonary disorders; known contraindication or hypersensitivity to etanercept or acitretin or their excipients; women who were pregnant, expecting to become pregnant, or breast-feeding during the study period; patients with any clinically relevant concurrent medical conditions such as active or chronic infections, including human immunodeficiency virus, hepatitis B virus and hepatitis C virus infections, and active or recent (within 2 years) tuberculosis; history of cancer (or carcinoma in situ) other than resected cutaneous basal cell or squamous cell carcinoma within the past 5 years before the screening visit; or patients who had received any investigational drug within 3 months of screening visit.

### Study design

**Fig. 1:**
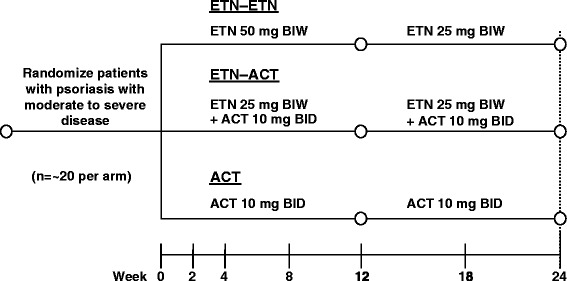
Study design. *ACT*, acitretin; *BID*, twice daily; *BIW*, twice weekly; *ETN*, etanercept

In this multicenter, randomized, open-label trial, patients were randomly assigned to one of three treatment groups: (a) etanercept 50 mg twice weekly (BIW) for 12 weeks followed by etanercept 25 mg BIW for a further 12 weeks (ETN–ETN); (b) etanercept 25 mg BIW and acitretin 10 mg twice daily (BID) for 24 weeks (ETN-ACT); (c) acitretin 10 mg BID for 24 weeks (ACT; Fig. 1). This study was conducted in compliance with the ethical principles of the Declaration of Helsinki and the International Conference on Harmonization Good Clinical Practice Guidelines and registered on ClinicalTrials.gov, identifier NCT00936065.

The primary endpoint was proportion of patients achieving ≥75 % improvement from baseline in PASI score (PASI 75) at week 24. Secondary endpoints included the proportion of patients achieving either PASI 75 or PASI 50 (≥50 % improvement from baseline) at each visit through week 24, PGA status of “clear/almost-clear”, and change in percent of BSA involvement from baseline over time.

### Statistical analysis

The sample size was calculated assuming 20 patients per group with a response rate of 35–65 % which would yield a confidence interval below ​± 21.9 %. Further assuming the response rate difference for primary efficacy endpoints is 20 % (i.e., 40 vs. 60 %), the 95 % confidence interval would be approximately ​± 30 %.

Statistical analysis was performed using SAS software, version 9.13. Efficacy evaluation was performed on the modified intent-to-treat (mITT) and per protocol (PP) population sets. The mITT population included all randomly assigned patients who received at least one dose of test medication and had both baseline and on-therapy PASI evaluations. The PP population included those members of the mITT population who had no major protocol violations that could potentially alter the interpretation of the efficacy analysis.

For the outcomes at each visit, the proportions of responders and the 95 % confidence interval (CI) were determined and the differences between treatment groups were assessed using Fisher exact test or Chi-square test with multiple comparisons, if necessary. Kaplan-Meier estimations for time to first occurrence of each event were determined and the log-rank test was used for statistical testing. For these analyses, no imputation was applied and the patients who did not experience the event were censored at the time of last observation.

Basic descriptive statistics are presented for other measures and the statistical significance of the change from baseline within the treatment groups was assessed using the paired *t*-test or the Wilcoxon signed-rank test. Differences in change from baseline between the treatment groups were assessed using one-way analysis of variance (ANOVA) or the Kruskal-Wallis test.

## Results

All results of this study have been posted on ClinicalTrials.gov, NCT00936065.

### Patients

**Fig. 2:**
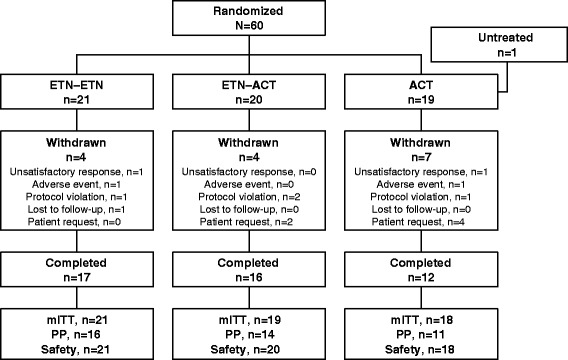
Patient disposition. *ACT*, acitretin; *ETN*, etanercept; *mITT*, modified intent-to-treat population; *PP*, per-protocol population

Of the 60 patients enrolled in this study and randomized to the three treatment arms, 45 completed the study (Fig. 2). One patient withdrew from the study after randomization but before receiving any study medication. Reasons for study discontinuation included patient request (*n* = 6), protocol violation (*n* = 4), unsatisfactory response (*n* = 2), adverse event (AE; *n* = 2), and lost to follow-up (*n* = 1). The baseline demographics were similar across all treatment arms (Table 1).

**Table 1:** Baseline demographics for the mITT population

Characteristic	ETN 50 mg–ETN 25 mg (*n* = 21)	ETN 25 mg–ACT 10 mg (*n* = 19)	ACT 10 mg (*n* = 18)	Total (*N* = 58)
Mean age, y (SD)	38.6 (9.5)	35.5 (8.8)	42.4 (12.0)	38.8 (10.3)
Mean height, cm (SD)	169.5 (9.3)	171.7 (5.5)	170.9 (6.8)	170.6 (7.4)
Mean weight, kg (SD)	74.1 (16.0)	74.0 (11.6)	74.2 (9.8)	74.1 (12.7)
Gender, n (%)				
Male	16 (76.2 %)	17 (89.5 %)	15 (83.3 %)	48 (82.8 %)
Cigarette status, n (%)				
Prior cigarette usage	5 (23.8 %)	5 (26.3 %)	3 (16.7 %)	13 (22.4 %)
Current cigarette usage	10 (47.6 %)	11 (57.9 %)	10 (55.6 %)	31 (53.5 %)
No	6 (28.6 %)	3 (15.8 %)	5 (27.8 %)	14 (24.1 %)
Alcohol status, n (%)				
Ex-drinker	2 (9.5 %)	2 (10.5 %)	2 (11.1 %)	6 (10.3 %)
Current-drinker	13 (61.9 %)	13 (68.4 %)	11 (61.1 %)	37 (63.8 %)
No	6 (28.6 %)	4 (21.1 %)	5 (27.8 %)	15 (25.9 %)
Prior therapies for psoriasis, n (%)				
Methotrexate	2 (9.5 %)	1 (5.3 %)	0	3 (5.2 %)
Cyclosporine	2 (9.5 %)	2 (10.5 %)	1 (5.6 %)	5 (8.6 %)
PUVA	0	3 (15.8 %)	0	3 (5.2 %)
Other^a^	12 (57.1 %)	12 (63.2 %)	10 (55.6 %)	34 (58.6 %)

*Abbreviations*: *ACT* acitretin, *CS* clinically significant, *ETN* etanercept, *mITT* modified intent-to-treat population, *NCS* not clinically significant, *PUVA* psoralen plus ultraviolet A radiation therapy, *SD* standard deviation

^a^Includes systemic antimycobacterials, medication for treating alimentary tract and metabolism conditions, cardiovascular drugs, respiratory drugs, dermatologicals, and systemic hormonal preparations

### Efficacy

**Fig. 3:**
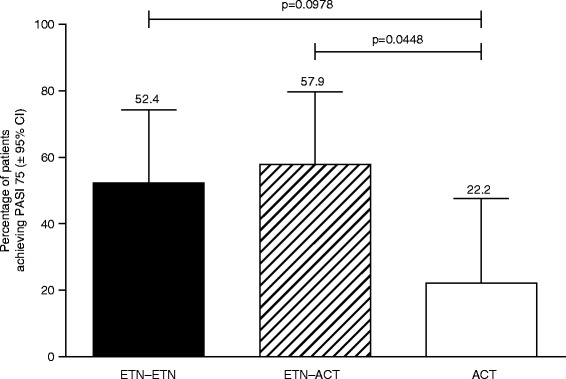
Proportion of patients achieving PASI 75 at week 24. *ACT*, acitretin; *ETN*, etanercept; *PASI*, Psoriasis Area Severity Index

The proportion of patients achieving PASI 75 by week 24 in the ETN–ETN and the ETN-ACT groups was numerically more than twice that observed for the ACT group (Fig. 3); however, only the pairwise comparison between the ETN-ACT and ACT groups showed statistical significance (*p* = 0.0448).

**Fig. 4:**
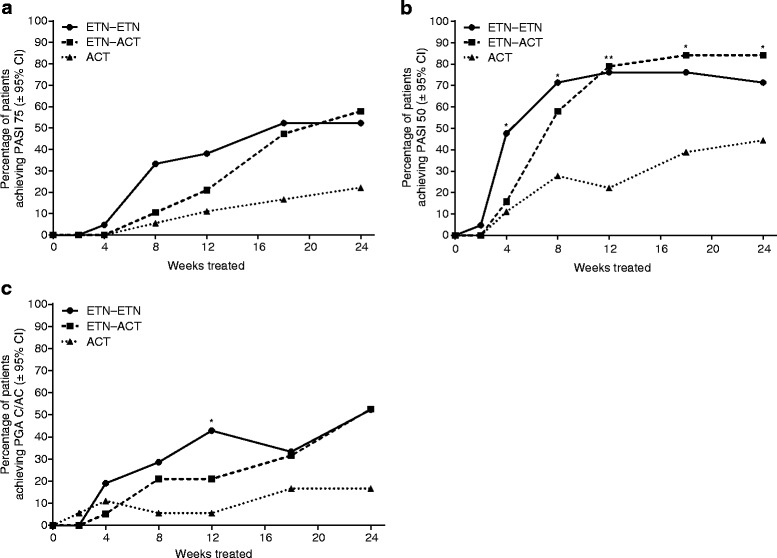
Proportion of patients achieving (**a**) PASI 75, (**b**) PASI 50, and (**c**) PGA clear/almost-clear at each visit. *ACT*, acitretin; *ETN*, etanercept; *PASI*, Psoriasis Area Severity Index; *PGA*, Physician’s Global Assessment. *p < 0.05; **p < 0.0005

The proportion of patients achieving PASI 75 increased at each visit for all treatment arms (Fig. 4a). Of the three treatment arms, patients in the ETN–ETN group demonstrated the greatest PASI 75 response during the first 18 weeks, after which their response was comparable to that observed for the ETN-ACT group. The PASI 75 responses demonstrated by both of these groups was numerically greater than double that observed for the ACT group. However, statistically significant treatment difference was not shown at any time point.

The proportion of patients achieving PASI 50 increased for all treatment arms (Fig. 4b). The change from baseline was statistically significant for all treatment arms. A greater proportion of patients in the ETN–ETN and ETN-ACT treatment arms achieved PASI 50 at all visits than did patients in the ACT arm.

The proportion of patients achieving PGA clear/almost-clear increased for all treatment arms (Fig. 4c). Patients in the ETN–ETN treatment arm demonstrated the greatest improvements up to week 12, after which improvements were similar to those of patients in the ETN-ACT treatment arm. The proportion of patients achieving PGA clear/almost-clear in the ETN–ETN and ETN-ACT arms was more than triple that for the ACT arm.

The median time to achieve PASI 75 for patients in the ETN–ETN arm was 126 days compared with 146 days for patients in the ETN-ACT arm (Table 2). The median time to achieve PASI 50 was the same for patients in the ETN–ETN and the ETN-ACT arms (56 days) and much shorter than for patients in the ACT arm (126 days). The difference was statistically significant among the three treatment arms (PASI 75: *p* = 0.0448 and PASI 50: *p* = 0.0033). Additionally, the median time to achieve PGA clear/almost-clear was comparable between the ETN–ETN and ETN-ACT treatment arms (167 and 165 days, respectively). The median time to achieve PASI 75 and PGA clear/almost-clear could not be determined for patients in the ACT arm.

**Table 2:** Median time to response

Response	Median time to response, days (95 % CI)			*p* value
	ETN 50 mg–ETN 25 mg (*n* = 21)	ETN 25 mg–ACT 10 mg (*n* = 19)	ACT 10 mg (*n* = 18)	
PASI 75	126 (56, 146)	146 (124, NA)	NA (127, NA)	0.0448
PASI 50	56 (28, 56)	56 (54, 84)	126 (56, NA)	0.0033
PGA clear/almost-clear	167 (55, 172)	165 (59, NA)	NA (87, NA)	0.3536

*Abbreviations*: *ACT* acitretin, *CI* confidence interval, *ETN* etanercept, *NA* not available, *PASI* Psoriasis Area Severity Index, *PGA* Physician’s Global Assessment

**Fig. 5:**
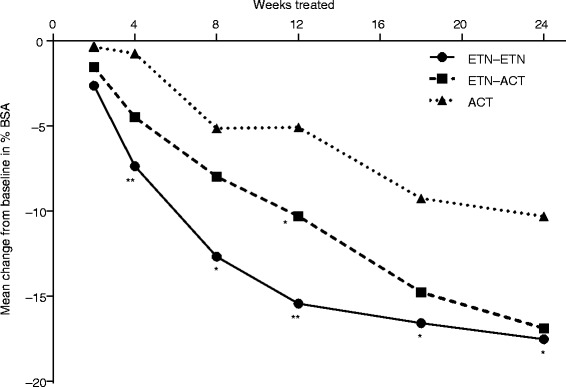
Change from baseline in percent BSA involvement of psoriasis at each visit. *ANCOVA,* analysis of covariance; *ACT*, acitretin; *BSA*, body surface area; *ETN*, etanercept. *p < 0.05; **p < 0.005 (treatment difference tested by ANCOVA)

After 24 weeks of treatment, mean reduction in percent BSA involvement from baseline was greatest in the ETN–ETN treatment arm (−17.5 %) compared with the ETN-ACT treatment arm (−16.9 %) and the ACT treatment arm (−10.3 %) at all visits (Fig. 5). The treatment difference was statistically significant at weeks 4, 8, 12, and 18, whereas the biggest reduction from baseline was at week 24 in all three treatment groups.

### Safety

Safety was evaluated on all patients who received at least one dose of study medication. Across the three treatment arms, 38 (64.4 %) patients reported 85 AEs, and 27 (45.8 %) patients reported 46 treatment-related AEs during the study. The overall incidence of AEs was similar in the three treatment arms (Table 3). The most common AEs reported across any treatment arm (Table 4) were pruritus (*n* = 6, 10.2 %), alopecia (*n* = 5, 8.5 %), and dry lips (*n* = 5, 8.5 %). One patient in the ACT arm reported a serious AE (severe back pain), which was determined by the investigator not to be related to the study treatment. No life-threatening treatment-emergent AEs occurred during the study nor were there any changes in laboratory tests, vital signs, or physical observations that were considered clinically important (Table 3).

**Table 3:** Summary of treatment-emergent adverse events

	All Causality TEAEs				Treatment-Related AE			
	ETN–ETN (*n* = 21)	ETN–ACT (*n* = 20)	ACT (*n* = 18)	Total (*N* = 59)	ETN–ETN (*n* = 21)	ETN–ACT (*n* = 20)	ACT (*n* = 18)	Total (*N* = 59)
Patients with TEAEs, n (%)	14 (66.7 %)	14 (70.0 %)	10 (55.6 %)	38 (64.4 %)	9 (42.9 %)	10 (50.0 %)	8 (44.4 %)	27 (45.8 %)
Total number of TEAEs	22	38	25	85	11	22	13	46
Patients with SAEs, n (%)	0 (0.0 %)	0 (0.0 %)	1 (5.6 %)	1 (1.7 %)	0 (0.0 %)	0 (0.0 %)	0 (0.0 %)	0 (0.0 %)
Total number of SAEs	0	0	1	1	0	0	0	0
Permanent discontinuation due to AE, n (%)	1 (4.8 %)	0	1 (5.6 %)	2 (3.4 %)	–	–	–	–

*Abbreviations*: *ACT* acitretin, *AE* adverse event, *ETN* etanercept, *SAE* serious adverse event, *TEAE* treatment-emergent adverse event

**Table 4:** Incidence of TEAEs in ≥10 % of patients in any treatment arm

System organ class	ETN 50 mg–ETN 25 mg (*n* = 21)	ETN 25 mg–ACT 10 mg (*n* = 20)	ACT 10 mg (*n* = 18)	Total (*N* = 59)
Skin and subcutaneous tissue disorders				
Pruritus	3 (14.3 %)	2 (10.0 %)	1 (5.6 %)	6 (10.2 %)
Alopecia	–	4 (20.0 %)	1 (5.6 %)	5 (8.5 %)
Skin exfoliation	–	2 (10.0 %)	1 (5.6 %)	3 (5.1 %)
Gastrointestinal disorders				
Dry lip	–	3 (15.0 %)	2 (11.1 %)	5 (8.5 %)
Cheilitis	–	2 (10.0 %)	2 (11.1 %)	4 (6.8 %)
Chapped lips	–	1 (5.0 %)	2 (11.1 %)	3 (5.1 %)
Investigations				
Alanine aminotransferase increased	1 (4.8 %)	2 (10.0 %)	–	3 (5.1 %)
Blood bilirubin increased	–	2 (10.0 %)	–	2 (3.4 %)
Musculoskeletal and connective tissue disorders				
Myalgia	–	–	2 (11.1 %)	2 (3.4 %)
Vascular disorders				
Hypertension	–	2 (10.0 %)	–	2 (3.4 %)

*Abbreviations*: *ACT* acitretin, *ETN* etanercept, *TEAE* treatment-emergent adverse event

## Discussion

Psoriasis is a chronic autoimmune disease and is often associated with joint inflammation in psoriatic arthritis, a comorbidity that affects between 10 and 30 % of all people with psoriasis [2, 3, 26]. Severe cases of psoriasis have been shown to affect health-related QoL to an extent similar to the effects of other chronic diseases such as depression, hypertension, congestive heart failure, or type 2 diabetes [27].

In multiple trials, etanercept has been shown to be effective in improving the disease severity of psoriasis and patient QoL [17–20]. However, treatment with high doses of etanercept can be expensive [28]. In this context, even though acitretin monotherapy is often only moderately effective [21–24], acitretin is widely used to treat psoriasis and is considered a standard of care in Korea. Furthermore, acitretin has been reported to have malignancy chemoprevention characteristics [21, 29]. Since acitretin is not an immunosuppressive agent and may act synergistically with biologic agents (e.g., etanercept), with a low risk of toxicity, a combination of etanercept and acitretin could be a viable treatment option for patients with psoriasis while keeping treatment affordable and potentially having additional beneficial effects. The results of a pilot study [25], a case series [30], and a posthoc review of clinical treatment practice [24] have suggested that the combination of etanercept and acitretin is effective and safe for the treatment of psoriasis. However, the efficacy and safety of this combination to treat Korean patients with psoriasis have not been determined until now.

The data presented here demonstrate that the combination of etanercept 25 mg BIW + acitretin 10 mg BID appears to be as effective for the treatment of psoriasis in Korean patients as etanercept 50 mg BIW for 12 weeks followed by etanercept 25 mg BIW. Furthermore, based on the numerical proportion of patients achieving efficacy endpoints (i.e., PASI 75, PASI 50, PGA clear/almost-clear, and reduction in disease-related BSA), and time to achieve these efficacy endpoints (i.e., time to achieve PASI 75, PASI 50, and PGA clear/almost-clear), both of these treatments appear to be more effective than acitretin 10 mg BID alone. In particular, the PASI 75 response rate at week 24 for the combined treatment arm (57.9 %) and the etanercept 50 mg–etanercept 25 mg arm (52.4 %) was numerically more than twice the rate of the acitretin 10 mg arm (22.2 %), although the differences were not statistically significant. However, unadjusted pairwise comparison showed a significant difference between the combined treatment group and acitretin 10 mg group (*p* = 0.0448). Furthermore, the proportion of patients achieving PGA clear/almost-clear in the ETN–ETN and ETN-ACT arms was more than triple that for the ACT arm. It is possible that due to the small number of patients, statistical significance was not achieved between the ETN–ETN and ETN-ACT arms and the ACT arm. Our results are consistent with an earlier Italian, randomized, controlled, pilot trial that showed PASI 75 response at week 24 was achieved by 45 % of the patients in the etanercept 25 mg BIW group, 44 % of the patients treated with etanercept 25 mg + acitretin, and 30 % of the patients in the acitretin 0.4 mg-kg^−1^ daily group. However, etanercept 25 mg BIW is not commonly effective in treating plaque psoriasis. Consequently, in our study, we treated patients at a higher initial dose of etanercept (50 mg BIW) and adjusted the dose downward to 25 mg BIW as the patients improved – a treatment regimen that we believe better reflects the real-world conditions.

These data demonstrate that the combination of etanercept with acitretin is just as effective as etanercept alone at week 24. However, patients treated with etanercept alone achieved the efficacy outcome endpoints faster than did patients receiving the combination. Treatment with etanercept alone may be preferred by patients wanting a more rapid resolution of their disease whereas treatment with the combination of these two drugs may be preferable for others without sacrificing efficacy or safety. The treating physician will need to consider these options in consultation with the patient to determine the optimum treatment regimen.

All treatments appeared to be safe and well tolerated by the patients. The incidence and severity of AEs appeared to be similar across all treatment arms. No life-threatening AEs were reported and no patient-reported SAEs that were related to study treatment. For patients with skin diseases, these results are important in terms of satisfaction with treatment and improvement of signs and symptoms of the disease, and could potentially affect their QoL.

## Conclusions

In this study, the treatment of psoriasis with the combination therapy of etanercept and acitretin substantially reduced the severity of disease in Korean patients over a period of 24 weeks. These results suggest that etanercept could be added to acitretin to treat Korean patients with psoriasis, especially when the disease is resistant or responding inadequately to topical treatment or phototherapy. Combination therapy of etanercept and acitretin, and etanercept alone, are both effective and well tolerated; however, the choice of treatment may depend on finding a balance between the costs of the treatment versus the rapidity with which patients desire to experience the benefits of the treatment.

## Abbreviations

ACT, acitretin; AE, adverse event; ANCOVA, analysis of covariance; ANOVA, analysis of variance; BID, twice daily; BIW, twice weekly; BSA, body surface area; CI, confidence interval; ETN, etanercept; mITT, modified intent-to-treat population; NA, not available; PASI, Psoriasis Area and Severity Index; PGA, Physician’s Global Assessment; PP, per-protocol population; PUVA, psoralen plus ultraviolet A radiation therapy; QoL, quality of life; SAE, serious adverse event; SD, standard deviation; TEAE, treatment-emergent adverse event; TNFα, tumor necrosis factor alpha

## Appendix

**Table:**

Site ID	Institution	PI	IRB No.
1002	Hallym University Medical Center	Kwang-Joong Kim	2010-S003
1003	The Catholic University of Korea Seoul St. Mary’s Hospital	Tae-Yoon Kim	KC09MSMV0185
1004	Samsung Medical Center	Joo-Heung Lee	2009-08-101
1005	Asian Medical Center	JeeHo Choi	2009-0645
1006	Bucheon St. Mary’s Hospital	ChulJong Park	HC09BSMV0091
1007	Konkuk University Hospital	YongBeom Choe	KUH1120010
1008	Korea University Medical Center, Guro Hospital	HaeJun Song	GR09163-001
1009	Kyung Hee University Medical Center	NackIn Kim	KMC IRB 0922-05
1010	Seoul National University Hospital	Jai-Il Yoon	H-0911-041-301
1011	Chungnam National University Hospital	JeungHoon Lee	0912-128
